# On Stammering

**Published:** 1831-01-01

**Authors:** 


### XXXVII.

### History of a Case of Stammering. By Dr. Bostock.

[Med.-Chir. Trans. Vol. XVI.]

Much has been written, and more has been promised, of late years, respecting the cure of stammering; and we ourselves have seen most beneficial effects result from a little artificial management in this disagreeable defect. But unfortunately, the benefits are seldom other than temporary; more, we firmly believe, owing to the inveteracy of habit and inattention, than to imperfection in the mode of treatment.

The case related by Dr. Bostock was that of a boy, who, at the age of two or three years, became suddenly and unaccountably affected with severe stammering. As the boy was plethoric, purgatives were prescribed, and as the effects appeared so favourable, they were often repeated, and still with temporary advantage. At length another auxiliary, or rather succedaneum, was tried—low diet, especially abstinence from animal food, and a very slender allowance of vegetables. This system was rigidly adhered to for several years, and the effects were sufficiently obvious in the boy's countenance and aspect. He became much less plethoric and florid; but was fully adequate to a participation in all the sports and exercises natural to his age. By a steady adherence to this discipline for about eight years, the complaint was kept at bay, but whenever any relaxation in the diet took place, or when the purgatives were omitted, or too long delayed, symptoms of the impediment immediately appeared. When about twelve years of age, the tendency to stammering seemed to be so far subdued, that a relaxation of the system was not followed by the usual consequences. Being put to a public school, where diet could not be so well attended to, a relapse took place, and proved extremely obstinate, so as to require a long and severe course of purgatives. The youth (now in his 15th year) may be said to be free from the complaint, and possesses a considerable volubility and rapidity of pronunciation. He has, however, occasional tendencies to relapse, when the bowels are disordered.

Some years ago, we had opportunities of seeing the means employed by two celebrated practitioners in the art of curing impediments of speech; and as the injunction of secrecy is no longer necessary to be observed, we shall state the principal means employed, and certainly with considerable success.

The fundamental principle is to fill the lungs with air, before the stammerer attempts to articulate—and the next step is to compress, by means of the voluntary muscles, the rima glottidis, so that the air, in rushing out, may make some sound; that is to say, the stammerer is to commence an expiration as if he were going to sing gently. If he now read or pronounce sentences, while the air is issuing through a narrowed channel, he will be surprised to find that no impediment to utterance occurs. By practising this mode of speaking or reading and never continuing to speak when the air is nearly exhausted, but always taking in a full inspiration and articulating with a narrow passage, he will soon have the command of speech. This constitutes, we may say, the entire secret, and we have seen people, in a few days, acquire uncommon facility in the delivery of their sentences. Unfortunately, there is such a perversity attending most stammerers, that they will not attend to the rules by which they were first cured, and most of them relapse into their former mal-habits. In one case which came under our painful notice for many years, any disordered state of the stomach or bowels invariably exasperated the stammering.

---

